# Supplementary material for: Uridine Derivatives: Synthesis, Biological Evaluation, and In Silico Studies as Antimicrobial and Anticancer Agents
Source: Medicina (Kaunas). 2023 Jun 7;59(6):1107. doi: 10.3390/medicina59061107 (PMC10302127; doi:10.3390/medicina59061107)
Supplement: Supplementary file 1 [file medicina-59-01107-s001.zip › medicina-2389379-supplementary.pdf]

medicina

Supplementary Materials

Fused Pyrimidine Derivatives: Synthesis, Biological Evaluation, and In Silico Studies as Antimicrobial and Anticancer Agents

Table S1. Name of the pathogenic microorganisms.

Types of Organisms	Strain	Reference
Gram-positive bacteria	*Bacillus subtilis*	ATCC 6633
	Bacillus cereus	BTCC 19
Gram-negative bacteria	*Escherichia coli*	ATCC 8739
	Salmonella typhi	AE 14612
	Pseudomonas aeruginosa	ATCC 9027
Name of the fungi	*Aspergillus niger*	ATCC 16404
	Aspergillus flavus	ATCC 204304

Table S2. The MIC and MBC values in mg/L of esters **4** and **5** against tested organisms.

Name of bacteria	MIC (mg/L)		MBC (mg/L)	
	Compound 4	Compound 5	Compound 4	Compound 5
E. coli	0.50	4.00	32.00	8.00
B. subtilis	4.00	0.50	8.00	8.00
B. cereus	0.13	1.00	32.00	16.00
S.typhi	0.50	0.50	16.00	8.00
P. aeruginosa	0.50	NF	16.00	NF

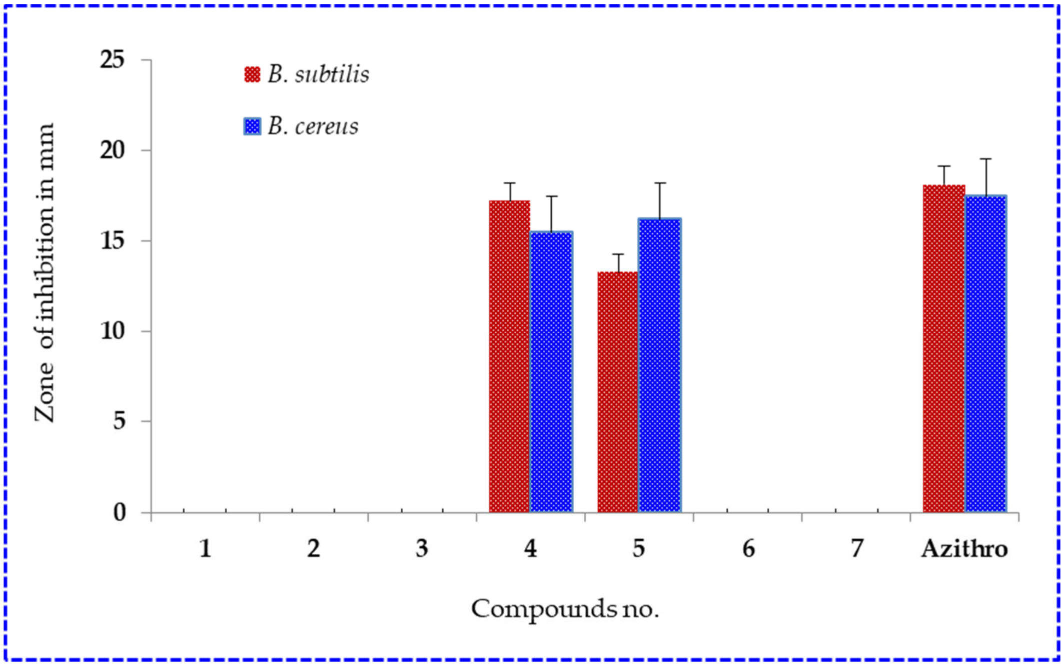

Figure S1. Zone of inhibition observed against Gram-positive bacteria by derivatives **2–7**.

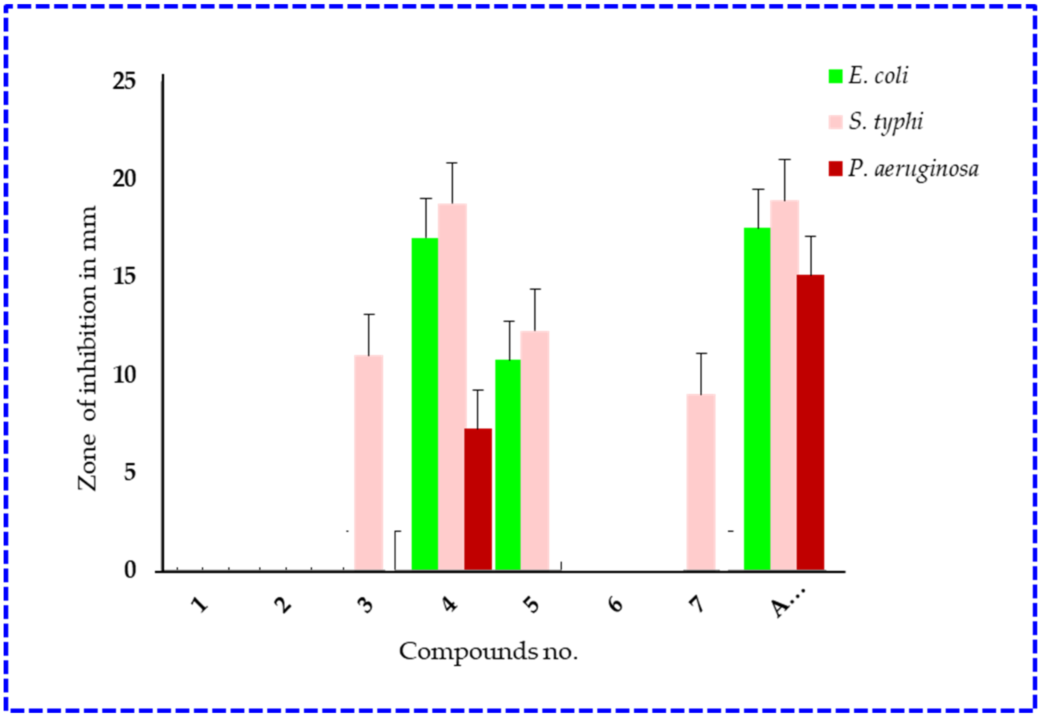

Figure S2. Zone of inhibition observed against Gram-negative bacteria by derivatives **2–7**.

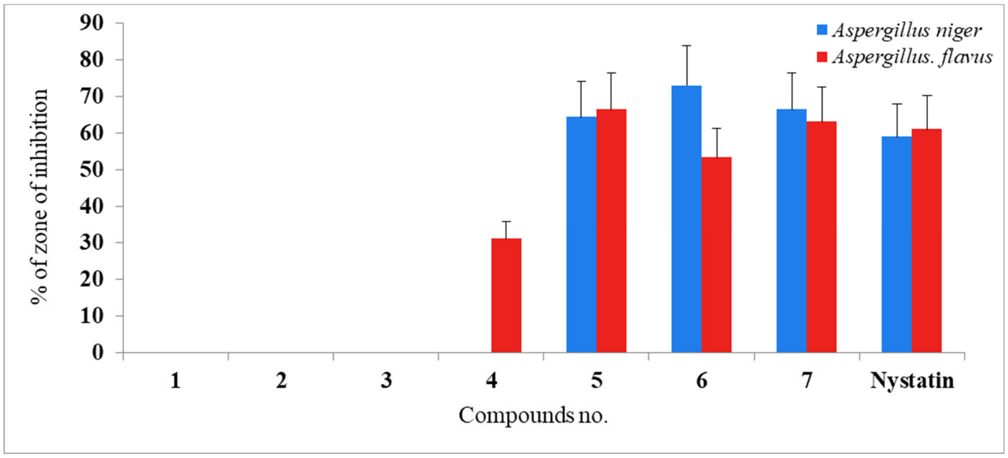

Figure S3. Antifungal activities of the synthesized uridine derivatives **2–7**.
